# Very Early Continence After Radical Prostatectomy and Its Influencing Factors

**DOI:** 10.3389/fsurg.2019.00060

**Published:** 2019-10-25

**Authors:** Lena Theissen, Felix Preisser, Mike Wenzel, Clara Humke, Frederik C. Roos, Luis A. Kluth, Andreas Becker, Severine Banek, Boris Bodelle, Jens Köllermann, Felix K. H. Chun, Philipp Mandel

**Affiliations:** ^1^Department of Urology, University Hospital, Goethe University, Frankfurt, Germany; ^2^Department of Radiology, University Hospital, Goethe University, Frankfurt, Germany; ^3^Senckenberg Institute of Pathology, University Hospital, Goethe University, Frankfurt, Germany

**Keywords:** early continence, radical prostatectomy, catheter removal, FFLU, nerve-sparing

## Abstract

**Introduction and Objectives:** Surgical techniques such as preservation of the full functional-length of the urethral sphincter (FFLU) have a positive impact on postoperative continence rates. Thereby, data on very early continence rates after radical prostatectomy (RP) are scarce. The aim of the present study was to analyze very early continence rates in patients undergoing FFLU during RP.

**Materials and Methods:** Very early-continence was assessed by using the PAD-test within 24 h after removal of the transurethral catheter. The PAD-test is a validated test that measures the amount of involuntary urine loss while performing predefined physical activities within 1 h (e.g., coughing, walking, climbing stairs). Full continence was defined as a urine loss below 1 g. Mild, moderate, and severe incontinence was defined as urine loss of 1–10 g, 11–50 g, and >50 g, respectively.

**Results:** 90 patients were prospectively analyzed. Removal of the catheter was performed on the 6th postoperative day. Proportions for no, mild, moderate and severe incontinence were 18.9, 45.5, 20.0, and 15.6%, respectively. In logistic regression younger age was associated with significant better continence (HR 2.52, *p* = 0.04), while bilateral nerve-sparing (HR 2.56, *p* = 0.057) and organ-confined tumor (HR 2.22, *p* = 0.078) showed lower urine loss, although the effect was statistically not significant. In MVA, similar results were recorded.

**Conclusion:** Overall, 64.4% of patients were continent or suffered only from mild incontinence at 24 h after catheter removal. In general, reduced urine loss was recorded in younger patients, patients with organ-confined tumor and in patients with bilateral nerve sparing. Severe incontinence rates were remarkably low with 15.6%.

## Introduction

Beside achieving adequate oncological outcomes, ensuring suitable functional outcomes, thus the preservation of erectile function and urinary continence, is a main goal after radical prostatectomy (RP). Postoperative incontinence still continues to be one of the most defacing side-effect, affecting about 9–16% of all patients after RP (1–4). The level of postoperative incontinence has an immense influence on patient's quality of life (1). For instance, people suffering from incontinence are more often afflicted with depression or sexual dysfunction (5).

Most commonly sphincter injury and bladder dysfunction are anatomically associated with postoperative urinary incontinence (6, 7). Therefore, improvement of surgical techniques is one of the main goals at RP to achieve improved functional postoperative outcomes (8). The urethral sphincter is part of an anatomic system complex including the pelvic floor muscles and its complex structures as well as several active and passive urethral closure mechanisms maintaining continence. Anatomically the functional urethra is partly located intraprostatically between the prostatic apex and the colliculus seminalis (9). Not only the preservation of its length although its anatomic fixation seems to be a determining factor to reach maximal continence (9). Hence, the preservation of the full functional-length of the urethral sphincter (FFLU) has been reported to positively impact postoperative continence at 12 months after RP (9).

Moreover, it has been reported, that the apical dissection during nerve sparing at RP itself improved long term continence rates as well as faster continence recovery (10). This said, the meticulous preparation of the urethra during FFLU and nerve-sparing RP may reduce the sphincter trauma and result in improved continence rates. Also patient characteristics [obesity = body mass index (BMI) ≥ 30] age at RR and tumor stage seem to have an influence on postoperative continence in general (11–13).

While data about the influence of preservation of FFLU on mid- and long-term continence rates is available, studies about very early continence rates (within 24 h after removal of the transurethral catheter) after RP are scant. Moreover, there is no consent about the impact of possible factors influencing very early continence such as BMI, age, nerve-sparing, and tumor stadium. In consequence, the aim of the present study was to report data on very early continence in patients undergoing FFLU and to analyze further influencing factors.

## Materials and Methods

In January 2018, the surgical concept of FFLU was implemented at our tertiary referral center. Data on very early continence was prospectively collected in patients with a sufficient postoperative cystogram on day 6 after RP undergoing RP with FFLU from January 2018 to March 2019. Patients were excluded from our analyses either because of missing data or because of an insufficient anastomosis in the postoperative cystogram at day 6. Ninety-four out of one hundred and fifty patients in the study period had their catheter removed at day 6 and underwent the PAD test. Four patients were excluded due to missing data, leaving 90 patients for the final analysis. Surgery was performed either with an open (*n* = 45, 50%) or robotically-assisted laparoscopic approach (*n* = 45, 50%). In all cases operation was performed by individualized apical preparation along patient's anatomic landmarks. Furthermore, the individual length of the external urethral sphincter was respected (9).

### Outcome

Very early continence was defined as continence within 24 h after removal of the transurethral catheter. To assess the rate of very early incontinence the so called PAD-test was used. The PAD-test is a comprehensible and validated test that measures the amount of involuntary loss of urine while performing predefined physical activities within 1 h (14, 15).

**Figure 1:**
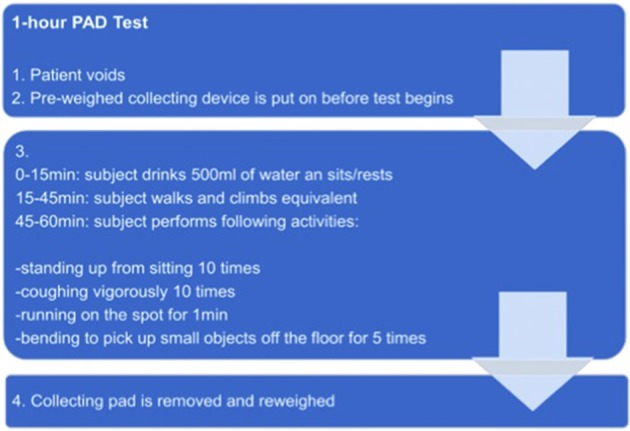
Procedure of PAD-test.

Before starting the test, patients void to empty the bladder completely. After putting on a pre-weighed collecting device, patients drink 500 ml of water and rest for 15 min. Afterwards, the patient has to move around for half an hour. In the following, patients are asked to perform several predefined physical activities within 15 min (e.g., coughing, walking, climbing stairs). At the end the collecting device is removed and reweighed (Figure 1).

The grade of continence is distinguished in different levels. Full continence is defined as an urine loss below 1 g. Mild, moderate and severe incontinence are defined as an urine loss of 1–10 g, 11–50 g, and >50 g, respectively (14, 15). In our analyses patients were stratified according to the different levels of continence/incontinence. Moreover, the influence of complete, partial or no intraoperative nerve-sparing, BMI, age, and pT-stage were assessed.

### Statistical Analysis

Descriptive statistics included frequencies and proportions for categorical coded variables. Medians and interquartile ranges (IQR) were reported for continuously coded variables. Uni- and multivariable logistic regression models were used to test the relationship between very early continence and patients' characteristics such as BMI, age, nerve-sparing, and tumor stadium. All tests were two sided with a level of significance set at *p* < 0.05. We used the statistical software Stata for our analysis (version 14 for Windows, StataCorp LP, College Station, TX).

## Results

Overall, 90 patients that underwent RP with FFLU were analyzed (Table 1). Median BMI was 26.0 kg/m^2^ and median age was 66.3 years. 62.2% of all patients had an organ-confined tumor. Bilateral/ unilateral nerve sparing was performed in 74.4%/23.3% of cases. Median loss of urine in PAD test was 5 g (Interquartile range 1–20 g). In general, very early continence rates were high in patients undergoing RP with FFLU. 18.9% of patients (*n* = 17) showed no incontinence 24 h after removal of the transurethral catheter. 45.5% (*n* = 41) of the sample suffered from mild incontinence. Moderate incontinence was documented in 20.0% of patients (*n* = 18), while severe incontinence occurred in 15.6% (*n* = 14). In sum, 64.4% of all patients (*n* = 58) were completely continent or showed only a mild form of incontinence at that early time-point after removal of the transurethral catheter.

**Table 1:** Patient characteristics.

Age median (IQR)	66.3 (60–70)
BMI median (IQR)	26.0 (24.4–28.6)
Nerve sparing	
no%	*n* = 2 (2.2%)
unilateral %	*n* = 21 (23.3%)
bilateral %	*n* = 76 (74.4%)
Organ-confined (%)	*n* = 56 (62.2%)
Loss of urine median (IQR)	5 g (1–20 g)
Continence (<1 g)	*n* = 17 (19%)
Mild incontinence (1–10 g)	*n* = 41 (45.5%)
Moderate incontinence (11–50 g)	*n* = 18 (20%)
Severe incontinence (<50 g)	*n* = 14 (15.5%)

In univariable logistic regression models predicting no or mild incontinence (<10 g) younger age (≤66 years) was significantly associated with lower loss of urine in PAD test (OR 2.52, 95%-CI: 1.03–6.17, *p* = 0.04). Conversely, obese patients showed similar continence rates in comparison to normal weighed patients (OR: 1.21, 95%-CI: 0.51–2.88, *p* = 0.7). Furthermore, bilateral nerve sparing and organ-confined pT-stage showed lower urine loss, although the effect was statistically not significant (HR: 2.56, *p* = 0.057 and HR: 2.22, *p* = 0.078, respectively). After adjustment in multivariable logistic models the results mentioned above remained qualitatively the same (Table 2). After adjustment for pT stage, nerve-sparing technique, age, and BMI, younger age remained an independent predictor for no or mild incontinence (OR: 2.83, 95%-CI: 1.08–7.43, *p* = 0.03).

**Table 2:** Uni- and multivariable logistic regression model predicting no or mild urine loss (≤10 g) in PAD test.

	**Univariable**			**Multivariable**		
	**OR**	**95%-CI**	***p*-value**	**OR**	**95%-CI**	***p*-value**
Age <66	2.52	1.03–6.17	0.043	2.83	1.08–7.43	0.03
Age ≥ 66	Ref.				Ref.	
pT2	2.22	0.91–5.40	0.078	2.57	0.98–6.71	0.05
≥pT3						
BMI < 26	1.21	0.51–2.88	0.66	1.07	0.42–2.73	0.88
BMI ≥ 26	Ref.				Ref.	
Bilateral NS	2.56	0.93–6.77	0.06	2.04	0.85–6.68	0.09
No/unilateral NS	Ref.				Ref.	

## Discussion

The preservation of continence after RP is an important aspect in the treatment of patients with prostate cancer. Postoperative incontinence has a strong influence of patient's quality of life (1). Several different factors like age, BMI, tumor stadium, or nerve sparing seem to have influence on postoperative continence rates. Nevertheless, data about their individual role and their individual impact on postoperative continence varies (11–13, 16, 17).

In general, a high level of postoperative continence and erectile function can be achieved nowadays (18–22). Recovery of continence has thereby to be seen as a process. While data on very early continence is rare, it is known that the majority of men are continent at month 3 after RP and recovery exceeds up to about 90% 1 year after RP and beyond (16, 17).

Therefore, the aim of the present study was to analyze continence rates within 24 h after removal of the catheter. To the best of our knowledge, no other study using a standardized and validated PAD test at this early timepoint is available. Our analysis demonstrated some noteworthy findings.

The surgical concept of FFLU shows high rates of very early continence. This is a novel finding as, almost all available data about postoperative continence rates focus rather on mid-or long term results. For example, Schlomm et al. showed that the preservation of the FFLU had a positive impact on postoperative continence rates. They compared continence rates 12 month after RP in patients with or without FFLU. Whereas, 50.1% of patient with FFLU were continent, continence rates were significantly worse in patients without FFLU (30.9%) 1 week after removal of the catheter (9). Twelve months after surgery patients with FFLU still showed better continence rates compared to patients operated without FFLU (96.9 and 94.7%). This finding is in concordance with our results, showing that continence rates 24 h after removal of the transurethral catheter are in general high in patients undergoing RP with FFLU. The only data available for very early continence (24 h after catheter removal) is reported without the FFLU technique and measured by PAD use rather than PAD test—and therefore are not comparable (23).

Furthermore, we could not show BMI to have an effect on very early continence. The influence of BMI on postoperative continence is controversially discussed in the literature. One study by Khoder et al. reported that obesity has a negative effect on postoperative continence at week 3 after surgery (12). In contrast Mandel et al. showed that obesity had no significant impact on postoperative continence 12 months after RP (11). Similar data, undermining that BMI has no significant influence on postoperative incontinence, comes from Mulholland et al. who examined the influence of BMI on continence after laparoscopic RP (24). Data about the influence of age on postoperative continence also varies. Zorn et al. concluded that younger men (<60 years) were significantly earlier continent compared to men older than ≥60 years at month 12 and 6 after RP. However, after 1 year of follow-up continence rates were similar among younger and older men (25). On the other hand, Labanaris et al. showed that selected men older than 75 years had excellent continence rates (at 12 months, ≤75 years 92.8% vs. ≥ 75 years 86.9%), although results were worse than in younger patients after robotic-assisted laparoscopic RP (26). In our study, age was the only significant factor influencing the very early continence. Younger patients (≤66 years) showed significant better continence rates compared to patients older than 66 years (*p* < 0.05).

There is still no consensus about the effect of preservation of neurovascular bundles (NB) on postoperative continence. In their study, Michl et al. showed that the level of postoperative incontinence depends on the surgical approach during RP, with or without initial nerve-sparing technique, not on the preservation of neurovascular bundles itself. Postoperative continence rates were almost the same in patients with preservation of the neurovascular bundles or with secondary resection, after initial neurovascular bundle preservation. In contrast, continence rates were significantly worse in patients with no nerve-sparing technique a priori (27). On the other hand, several studies show that nerve sparing itself has a positive effect on postoperative continence. For example Kim et al. showed that continence rates were significantly higher in patients with bilateral than in patients with unilateral or no nerve-sparing 1 year after surgery (28, 29). Even patients with preoperative erectile dysfunction showed better postoperative continence rates when nerve-sparing was performed (30). Our study undermines the importance of (bilateral) nerve-sparing showing a positive effect, despite in a non-significant fashion, on postoperative continence rates also at a very early timepoint.

The current study has several limitations. One limitation is the lack of control group of patients without FFLU. This would help to analyze the impact of FFLU on very early continence rates in more detailed fashion. Furthermore, our study lacks long-term follow-up to compare very early and long-term continence rates. Additionally it is difficult to conclude that factors affecting mid-and long-term continence rates might automatically have a similar influence on very-early continence. Moreover, only patients with catheter removal at day 6 postoperative were included in our study. This might lead to some kind of selection bias. Last but not least, all surgeries were performed by high-volume surgeons, which might have affected our results and may vary from other institutions. Nevertheless, to the best of our knowledge, this is the first study evaluating very early continence after RP with FFLU.

In summary, very early continence rates within 24 h after removal of the catheter are high in patients undergoing RP with FFLU. 64.4% of patients were continent or suffered only from mild incontinence at that early time-point after RP. Age was an independent predictor for incontinence. In general, a reduced urine loss was recorded in younger patients, patients with organ-confined tumors and in patients with bilateral nerve sparing during RP (the last two with statistically insignificant effects). Severe incontinence rates were remarkably low with 15.6%. Nevertheless, a larger patient cohort with longer follow-up is needed to correlate the results of very-early continence with long-term continence.

## Data Availability Statement

All datasets generated for this study are included in the manuscript/supplementary files.

## Ethics Statement

The study was approved by the institutional review boards of the University Cancer Centre Frankfurt and the Ethical Committee at the University Hospital Frankfurt. All patients included in our study signed a written informed consent.

## Author Contributions

CH and MW: protocol development, acquisition of the analyzed data. FP: data analysis. FR, AB, SB, LK, JK, and BB: acquisition of analyzed data, contribution to the conception of work, final approval of manuscript. FC: substantial contributions to the conception of the work, critical revision of intellectual content. PM: substantial contributions to the conception of work, critical revision of work, data analysis, and final approval of the version to be published. LT: protocol development, acquisition of the analyzed data, analysis and interpretation of data, substantial contributions to the conception of work, drafting the work, and revising the work for the intellectual content. All authors complied with all aspects of the work. They ensure that questions related to the accuracy of the work are adequately discussed and solved.

### Conflict of Interest

The authors declare that the research was conducted in the absence of any commercial or financial relationships that could be construed as a potential conflict of interest.

## References

[1] Radadia KD Farber NJ Shinder B Polotti CF Milas LJ Tunuguntla HSGR. Management of postradical prostatectomy urinary incontinence: a review. Urology. (2018) 113:13–9. 10.1016/j.urology.2017.09.025 29031841

[2] Mottet N van den Bergh R Briers E Cornford P Santis MD Fanti S EAU Guidelines on Prostate Cancer. Arnhem: European Association of Urology (2019).

[3] Yuh B Wilson T Bochner B Chan K Palou J Stenzl A. Systematic review and cumulative analysis of oncologic and functional outcomes after robot-assisted radical cystectomy. Eur Urol. (2015) 67:402–22. 10.1016/j.eururo.2014.12.008 25560797

[4] Mandel P Greafen M Michl U Huland H Tilki D. The effect of age on functional outcomes after radical prostatectomy. Urol Oncol. (2015) 33:203.e11–8. 10.1016/j.urolonc.2015.01.015 25814146

[5] Sarikaya S Yildiz FG Senocak C Bozkurt OF Karatas OF Urinary incontinence as a cause of depression and sexual dysfunction: questionnaire-based study. Rev Int Androl. (2018) 18:30082–7. 10.1016/j.androl.2018.08.003 30470663

[6] Hammerer P Huland H. Urodynamic evaluation of changes in urinary control after radical retropubic prostatectomy. J Urol. (1997) 157:233–36. 8976260

[7] Doudt A Zuckerman J. Male slings for post-prostatectomy incontinence. Rev Urol. (2018) 20:158–69. 10.3909/riu080 30787674 PMC6375003

[8] Sridhar AN Abozaid M Rajan P Sooriakumaran P Shaw G Nathan S. Surgical techniques to optimize early urinary continence recovery post robot assisted radical prostatectomy for prostate cancer. Curr Urol Rep. (2017) 18:71. 10.1007/s11934-017-0717-4 28718165 PMC5514172

[9] Schlomm T Heinzer H Steuber T Salomon G Engel O Michl U. Full functional-length urethral sphincter preservation during radical prostatectomy. Eur Urol. (2011) 60:320–9. 10.1016/j.eururo.2011.02.040 21458913

[10] Ludovico GM Dachille G Pagliarulo G D'Elia C Mondaini N Gacci M Bilateral nerve sparing robotic-assisted radical prostatectomy is associated with faster continence recovery but not with erectile function recovery compared with retropubic open prostatectomy: the need for accurate selection of patients. Oncol Rep. (2013) 29:2445–50. 10.3892/or.2013.2365 23545628

[11] Mandel P Kretschmer A Chandrasekar T Nguyen HG Buchner A Stief CG. The effect of BMI on clinicopathologic and functional outcomes after open radical prostatectomy. Urol Oncol. (2014) 32:297–302. 10.1016/j.urolonc.2013.09.005 24332640

[12] Khoder WY Trottmann M Stuber A Stief CG Becker AJ. Early incontinence after radical prostatectomy: a community based retrospective analysis in 911 men and implications for preoperative counseling. Urol Oncol. (2013) 31:1006–11. 10.1016/j.urolonc.2011.10.003 22100069

[13] Tilki D Maurer V Pompe RS Chun FK Preisser F Haese A. Tumor characteristics, oncological and functional outcomes after radical prostatectomy in very young men ≤ 45 years of age. World J Urol. (2019). 10.1007/s00345-019-02740-8. [Epub ahead of print]. 30937571

[14] Hahn I Fall I Objective quantification of stress urinary incontinence: a short, reproducible, provocative pad-test. Neurourol Urodyn. (1991) 10:475–81. 10.1002/nau.1930100503

[15] Kralj D Comparative study of pad tests- reliability and repetitivness. Neurourol Urodyn. (1989) 8:305–06.

[16] Kuehhas FE Naegele R Eckersberger E Margreiter M Herwig R Kazzazi A. Urinary continence after radical prostatectomy: the patient perspective. Can J Urol. (2011) 18:5811–8. 21854713

[17] Zorn KC Wille MA Thong AE Katz MH Shikanov SA Razmaria A. Continued improvement of perioperative, pathological and continence outcomes during 700 robot-assisted radical prostatectomies. Can J Urol. (2009) 16:4742–9. 19671227

[18] Alenizi AM Zorn KC Bienz M Rajih E Hueber PA Al-Hathal N. Erectile function recovery after robotic-assisted radical prostatectomy (RARP): long term exhaustive analysis across all preoperative potency categories. Can J Urol. (2016) 23:8451–56. 27705730

[19] Cathala N Mombet A Sanchez-Salas R Rozet F Barret E Giuliano F. Evaluation of erectile function after laparoscopic radical prostatectomy in a single center. Can J Urol. (2012) 19:6328–35. 22892254

[20] Johnson ME Zaorsky NG Martin JM Ruth K Greenberg RE Uzzo RG. Patient reported outcomes among treatment modalities for prostate cancer. Can J Urol. (2016) 23:8535–45. 27995848

[21] Lepor H Kaci L. The impact of open radical retropubic prostatectomy on continence and lower urinary tract symptoms: a prospective assessment using validated self-administered outcome instruments. J Urol. (2004) 171:1216–9. 10.1097/01.ju.0000113964.68020.a7 14767305

[22] Mandel P Preisser F Graefen M Steuber T Salomon G Haese A. High chance of late recovery of urinary and erectile function beyond 12 months after radical prostatectomy. Eur Urol. (2017) 71:848–50. 10.1016/j.eururo.2016.09.030 27743754

[23] Manfredi M Checcucci E Fiori C Garrou D Aimar R Amparore D. Total anatomical reconstruction during robot-assisted radical prostatectomy: focus on urinary continence recovery and related complications after 1000 procedures. BJU Int. (2019) 124:477–86. 10.1111/bju.14716 30801887

[24] Mulholland TL Huynh PN Huang RR Wong C Diokno AC Peters KM Urinary incontinence after radical retropubic prostatectomy is not related to patient body mass index. Prostate Cancer Prostatic Dis. (2006) 9:153–9. 10.1038/sj.pcan.4500860 16505832

[25] Zorn KC Mendiola FP Rapp DE Mikhail AA Lin S Orvieto MA. Age-stratified outcomes after robotic-assisted laparoscopic radical prostatectomy. J Robot Surg. (2007) 1:125–32. 10.1007/s11701-007-0009-y 25484948 PMC4247449

[26] Labanaris AP Witt JH Zugor V. Robotic-assisted radical prostatectomy in men ≥75 years of age. Surgical, oncological and functional outcomes. Anticancer Res. (2012) 32:2085–9. 22593493

[27] Michl U Tennste P Feldmeier E Mandel P Oh SJ Ahyai S Nerve-sparing surgery technique, not the preservation of the neurovascular bundles, leads to improved long-term continence rates after radical prostatectomy. Eur Urol. (2016) 69:584–9. 10.1016/j.eururo.2015.07.037 26277303

[28] Kim M Park M Pak S Choi SK Shim M Song C. Integrity of the urethral sphincter complex, nerve-sparing, and long-term continence status after robotic-assisted radical prostatectomy. Eur Urol Focus. (2018) 5:823–30. 10.1016/j.euf.2018.04.021 29759661

[29] Wang X Wu Y Guo J Chen H Weng X Liu X. Intrafascial nerve-sparing radical prostatectomy improves patients' postoperative continence recovery and erectile function: a pooled analysis based on available literatures. Medicine. (2018) 97:e11297. 10.1097/MD.0000000000011297 30024505 PMC6086530

[30] Khoder WY Waidelich R Seitz M Becker AJ Buchner A Trittschler S. Do we need the nerve sparing radical prostatectomy techniques (intrafascial vs. interfascial) in men with erectile dysfunction? Results of a single-centre study. World J Urol. (2015) 33:301–7. 10.1007/s00345-014-1302-9 24752607

